# Lower Circulating B12 Is Associated with Higher Obesity and Insulin Resistance during Pregnancy in a Non-Diabetic White British Population

**DOI:** 10.1371/journal.pone.0135268

**Published:** 2015-08-19

**Authors:** Bridget Ann Knight, Beverley M. Shields, Adam Brook, Anita Hill, Dattatray S. Bhat, Andrew T. Hattersley, Chittaranjan S. Yajnik

**Affiliations:** 1 Exeter NIHR Clinical Research Facility, University of Exeter Medical School, Exeter, England; 2 Royal Devon and Exeter NHS Foundation Trust, Exeter, England; 3 Diabetes Unit, KEM Hospital and Research Centre, Pune, India; CSIR-INSTITUTE OF GENOMICS AND INTEGRATIVE BIOLOGY, INDIA

## Abstract

**Objective:**

Vitamin B12 and folate are critical micronutrients needed to support the increased metabolic demands of pregnancy. Recent studies from India have suggested that low vitamin B12 and folate concentrations in pregnancy are associated with increased obesity; however differences in diet, antenatal vitamin supplementation, and socioeconomic status may limit the generalisability of these findings. We aimed to explore the cross-sectional relationship of circulating serum vitamin B12 and folate at 28 weeks’ gestation with maternal adiposity and related biochemical markers in a white non diabetic UK obstetric cohort.

**Methods:**

Anthropometry and biochemistry data was available on 995 women recruited at 28 weeks gestation to the Exeter Family Study of Childhood Health. Associations between B12 and folate with maternal BMI and other obesity-related biochemical factors (HOMA-R, fasting glucose, triglycerides, HDL and AST) were explored using regression analysis, adjusting for potential confounders (socioeconomic status, vegetarian diet, vitamin supplementation, parity, haemodilution (haematocrit)).

**Results:**

Higher 28 week BMI was associated with lower circulating vitamin B12 (r = -0.25; P<0.001) and folate (r = -0.15; P<0.001). In multiple regression analysis higher 28 week BMI remained an independent predictor of lower circulating B12 (β (95% CI) = -0.59 (-0.74, -0.44) i.e. for every 1% increase in BMI there was a 0.6% decrease in circulating B12). Other markers of adiposity/body fat metabolism (HOMA-R, triglycerides and AST) were also independently associated with circulating B12. In a similar multiple regression AST was the only independent obesity-related marker associated with serum folate (β (95% CI) = 0.16 (0.21, 0.51))

**Conclusion:**

In conclusion, our study has replicated the previous Indian findings of associations between lower serum B12 and higher obesity and insulin resistance during pregnancy in a non-diabetic White British population. These findings may have important implications for fetal and maternal health in obese pregnancies.

## Introduction

The rise in BMI in the general population is reflected in the more than doubling (7.6% to 15.6%) over the last 20 years of the proportion of women who are obese at the time of booking with the maternity services[1]. These rising rates of obesity in pregnancy have led to an emphasis on understanding the effects of adiposity on maternal health. The paradox of nutritional deficiencies in those who are overweight and obese[2] may have important implications for maternal and fetal health and wellbeing in pregnancy.

The micronutrients B12 and folate are needed to support the increased demands of the fetus in pregnancy. Both vitamins are involved in one-carbon metabolism and methylation processes, including epigenetic modulation and DNA synthesis and repair, assisting the normal processes of fetal growth and development. Deficiency of these vitamins is associated with wide-ranging multi-system abnormalities, including megaloblastic anaemia in the mother, and growth disturbances, birth defects and neurocognitive disorders in the offspring[3].

Lower B12 in pregnancy was associated with higher maternal BMI and/or increased offspring insulin resistance in a series from India[4–6], Given the differences in diet, antenatal vitamin supplementation, and socioeconomic status, replication in a Western setting will help generalisation of the finding.

The aim of our study was to investigate this relationship between maternal BMI and serum vitamin B12 and folate in a UK pregnancy cohort. We studied the associations between circulating serum B12 and folate concentrations and measures of obesity and related glycaemic and metabolic biomarkers measured at 28 weeks gestation in non-diabetic, singleton, white pregnancies.

## Methods

### Subjects

Anthropometric measurements and biochemical data were available on 995 women recruited as part of the Exeter Family Study of Childhood Health[7]. Height, weight, and fasting bloods were taken at 28 weeks of pregnancy (+/-5 days). Routine analysis for fasting plasma glucose (FPG) was undertaken by the pathology labs at the Royal Devon and Exeter NHS Foundation Trust, UK (women with FPG >5.5 mmol/l were excluded from analysis). Serum insulin was measured at the University Hospital Birmingham NHS Trust, UK using an immunochemiluminometric assay (Molecular Light Technology, Cardiff, UK). The assay is specific for insulin and the interassay coefficients of variation were less than 9.0% over the concentration range reported. Socio-economic status (SES) was based on post-code and determined by Townsend deprivation score. Insulin resistance was calculated using the homeostatic model assessment (HOMA)[8].

### B12 and folate measurements

B12 and folate were measured in serum samples taken at 28 weeks of pregnancy, and stored at -80°C. B12 and folate were analysed with microbiological assays at the Diabetes Research Centre, KEM Hospital, Pune, India. Intra/inter-assay coefficients of variation were <8% for both assays. Low B12 was defined as serum B12 <150 pmol/l and low folate as <7 nmol/l, as previously described[5].

### Statistics

Variables were assessed for normality and log transformations used where appropriate. Geometric means and standard deviation (SD) range (+/- 1SD on the log scale, then back-transformed) are therefore presented for these variables. T-tests were used to compare means. Natural log transformation was used to aid interpretation of beta coefficients in regression analysis (allowing regression coefficients to be interpreted in terms of percentage change[9]). Pearson correlation coefficients were used to assess associations between the B12 and folate results, and the measures of obesity and other metabolic parameters. Regression analysis was used to explore the associations of BMI and obesity-related factors with B12 and folate, whilst adjusting for potential confounders.

### Ethics

All participants gave written informed consent. Ethical approval was granted by the North and East Devon Research Ethics Committee.

## Results

### Baseline characteristics (Table 1)

**Table 1:** Baseline characteristics of study cohort (n = 995) at 28 weeks gestation.

Variables	Mean +/- SD or*Geometric mean (SD range)
Micronutrient concentrations	
- Serum B12 (pmol/L)*	204.2 (104.3–295.1)
- Serum Folate (nmol/L)*	14.5 (7.6–27.5)
Anthropometric measures	
- BMI(kg/m^2^)*	27.5 (23.4–32.4)
Lifestyle/Demographics	
- Age(yrs)	30.4 +/- 5.2
- Socio Economic Status (Townsend score)	0.3 +/- 3.1
- Smoking (%)	13.7
- Vegetarian (%)	8.5
- Multivitamin during pregnancy (%)	30
Pregnancy related factors	
- Primiparity (%)	45.9
Haemodilution	
- Haematocrit	34.1 +/- 2.6
BMI associated metabolic factors	
- HOMA-R*	1.0 (0.8–2.2)
- Triglycerides (mmol/L)	2.0(1.5–2.8)
- HDL cholesterol	2.1 +/- 0.5
- AST (mmol/L)*	17.8 (13.8–22.9)
- FPG (mmol/L)	4.4 +/- 0.4

Data presented as mean +/- SD (or *geometric mean (SD range) for log transformed data). Discrete variables presented as (%) for characteristic.

AST = Aspartate Aminotransferase.

995 women were recruited at 28 weeks of pregnancy. 26.4% had a BMI ≥30kg/m^2^, 54.1% were multiparous and 13.7% reported smoking in pregnancy. 90% of mothers took folic acid supplements in early pregnancy (until 12 weeks gestation), 30% continued to take a multivitamin during pregnancy and 8.5% were vegetarian.

### Serum B12 and folate concentrations in pregnancy

The geometric mean (SD range) serum B12 concentration was 204.2 (141.3–295.1) pmol/l and 20% of the cohort had B12 <150 pmol/l. The geometric mean (SD range) serum folate concentration was 14.5 (7.6–27.5) nmol/l, and 14.7% of the cohort had folate concentrations <7 nmol/l. B12 concentration was positively correlated with folate concentration (r = 0.221, p<0.001)

### Higher maternal BMI was associated with lower B12 and folate

Higher BMI at 28 weeks pregnancy was associated with lower circulating serum vitamin B12 and folate (r = -0.252 and -0.151 respectively; P<0.001 for both). (Table 2)

**Table 2:** Univariate associations between B12, folate and BMI with potential confounders (lifestyle/demographic, pregnancy related and haemodilutional factors) in 995 pregnant women. Pearson correlation coefficients (r) presented for continuous variables

Factor	B12	Folate*	28 week BMI*
	r (p)	r (p)	r (p)
BMI (kg/m^2^)*	-0.25 (<0.001)	-0.15 (<0.001)	-
Lifestyle/demographic			
- Age	0.09 (0.007)	0.20 (<0.001)	-0.07 (0.02)
- Townsend Score	-0.08 (0.008)	-0.09 (0.003)	0.08 (0.009)
- Smoking	-0.12 (<0.001	-0.22 (<0.001)	0.07 (0.026)
- Vegetarianism	-0.01 (0.7)	0.24 (<0.001)	-0.03 (0.3)
- Vitamin use	0.18 (<0.001)	0.50 (<0.001)	-0.07 (0.025)
Pregnancy related			
- Parity	0.07 (0.038)	0.18 (<0.001)	-0.03 (0.3)
Haemodilutional			
- Haematocrit (%)	0.15 (<0.001)	0.08 (0.01)	0.08 (0.018)

* denotes log transformed data

### Could these associations be explained by other factors?

To analyse potential confounders, the effects of maternal lifestyle factors, pregnancy factors, and haemodilution, on vitamin B12 and folate were examined (Table 2)

#### B12 associations

In a univariate analysis lower B12 was seen in younger mothers and those with higher social deprivation. B12 was higher in women who had taken vitamin supplements during pregnancy (224 vs 195 pmol/l, p<0.001). There was no difference in B12 concentrations between women who were vegetarian and those who were non-vegetarian (199. vs 204pmol/l, p = 0.7). Haematocrit (a marker of ‘haemodilution’ in pregnancy) was positively associated with B12 concentrations.

In a multiple regression analysis (Table 3), BMI, vitamin supplements and haematocrit were the only factors that remained significantly associated with B12. Lower B12 concentrations were associated with higher BMI, independently of maternal demographic and lifestyle factors, pregnancy related factors, and haemodilution (Hct): Log BMI: β (95%CI) = -0.59 (-0.74, -0.44), p<0.001). This suggests for every 1% increase in BMI there will be a 0.6% decrease in circulating B12.

**Table 3:** Multiple regression analysis to demonstrate the relationships between BMI and potential confounding variables on serum vitamin B12. Partial R is the adjusted correlation coefficient.

Factors associated with serum vitamin B12*	Partial R	β	95% CI for β	t	p
BMI (kg/m2)*	-0.25	-0.59	(-0.74, -0.44)	7.75	<0.001
Social/demographic					
- age	0.03	0.002	(-0.003,0.007)	0.77	0.4
- Townsend score	-0.03	-0.003	(-0.01, 0.004)	-0.76	0.5
- smoking	-0.05	-0.06	(-0.13, 0.01)	-1.59	0.1
- vegetarianism	-0.03	-0.04	(-0.12, 0.04)	-0.93	0.4
- Vitamin use	0.14	0.11	(0.06, 0.16)	4.16	<0.001
Pregnancy related					
- Parity	0.01	0.009	(-0.04, 0.06)	0.37	0.7
Haemodilutional					
- Haematocrit (%)	0.15	2.11	(1.21, 3.01)	4.59	<0.001

*Denotes variable transformed using natural logs (allowing β coefficients to be interpreted in terms of percentage change)

#### Folate associations

In univariate analysis, lower folate was seen in younger mothers and those with higher social deprivation scores. Folate concentrations were higher in women who had taken vitamin supplements during pregnancy (22.9 v 12.0 nmol/l, p<0.001) and in vegetarians (23.4 v 13.8 nmol/l, p<0.001). Haematocrit (a marker of ‘haemodilution’ in pregnancy) was positively associated with maternal folate concentrations (Table 2)

In a multiple regression analysis (Table 4) vegetarian diet, age, BMI, vitamin supplements and parity remained significantly associated with folate. Lower folate concentrations were associated with higher BMI, independently of these factors: Log BMI β (95%CI) = -0.42 (-0.65, -0.19), p<0.001). This suggests for every 1% increase in BMI there will be a 0.4% decrease in circulating folate.

**Table 4:** Multiple regression analysis to demonstrate the relationships between BMI and potential confounding variables on serum folate. Partial R is the adjusted Pearson correlation coefficient.

Factors associated with serum folate*	Partial R	Beta	95% CI	t	p
BMI (kg/m2)*	-0.12	-0.42	(-0.65, -0.19)	-3.63	<0.001
Social/demographic					
- Age	0.16	0.02	(0.01, 0.03)	4.91	<0.001
- Townsend score	-0.008	-0.001	(-0.01, 0.01)	-0.23	0.8
- Smoking	-0.09	-0.16	(-0.27, -0.05)	-2.84	0.005
- Vegetarianism	0.18	0.36	(0.23, 0.49)	5.53	<0.001
- Vitamin use	0.41	0.54	(0.47, 0.62)	13.39	<0.001
Pregnancy related					
- Parity	0.12	0.14	(0.07, 0.22)	3.72	<0.001
Haemodilutional					
- Haematocrit (%)	0.07	1.58	(0.20, 2.96)	2.25	0.025

* Denotes variable transformed using natural logs (allowing β coefficients to be interpreted in terms of percentage change)

### Which factors associated with obesity explain the variation in B12 and folate measurements?

In an attempt to understand potential mechanisms explaining the association between BMI, and B12 and folate, we added other obesity-related factors (HOMA-R, fasting glucose, triglycerides, HDL and AST) to the model (Table 5).

**Table 5:** Pearson correlation coefficients (r) to demonstrate the univariate associations between B12, folate and BMI with obesity related metabolic factors. Pearson correlation coefficients (r) presented for continuous variables

Factor	B12*	Folate*	28 week BMI
	r (p)	r (p)	r (p)
Obesity related metabolic factors			
- HOMAR*	-0.22 (<0.001	-0.16 (<0.001)	0.53 (<0.001)
- FPG	-0.09 (0.006)	-0.04 (0.3)	0.30 (<0.001)
- Triglycerides*	-0.21 (<0.001	-0.02 (0.5)	0.24 (<0.001)
- HDL	0.15 (<0.001)	0.14 (<0.001)	-0.11 (<0.001)
- AST*	0.31 (<0.001)	0.23 (<0.001)	-0.16 (<0.001)

*denotes log transformed data

Table 5 shows the univariate associations of other obesity related factors with BMI, B12 and folate. BMI was positively associated with HOMA-R, FPG, and Triglycerides, and negatively with HDL and AST. B12 was negatively associated with HOMA-R, FPG, and Triglycerides and positively with HDL and AST. Associations with folate were weaker than those with B12, with only HOMA-R, HDL and AST remaining statistically significant.

#### B12 associations

In multiple regression analysis, the association between B12 and BMI remained significant when adding in other obesity-related factors. HOMA-R, triglycerides, and AST also explained variation in B12, independently of BMI (Table 6)).

**Table 6:** Multiple regression analysis to demonstrate the relationships between BMI, BMI associated factors, confounding variables and, haemodilution on serum vitamin B12. Partial R is the adjusted pearson correlation coefficient.

Factors associated with serum vitamin B12*	Partial R	Beta	95% CI	t	p
BMI (kg/m2)*	-0.15	-0.391	(-0.57, -0.21)	-4.30	<0.001
BMI associated factors					
- HOMAR*	-0.09	-0.08	(-0.14, -0.02)	-2.53	0.01
- Triglycerides (mmol/L)*	-0.11	-0.12	(-0.20, -0.05)	-3.20	0.001
- HDL Cholesterol	0.015	0.012	(-0.04, 0.06)	0.45	0.6
- AST (mmol/L)*	0.21	0.31	(0.22, 0.40)	6.4	<0.001
- FPG	0.024	0.025	(-0.04, 0.09)	0.71	0.48
Social/demographic					
- Age	-0.003	0	(-0.005, 0.005)	0.1	0.9
- Townsend score	-0.014	-0.002	(-0.009, 0.006)	-0.41	0.7
- smoking	-0.011	-0.01	(-0.09, 0.06)	0.03	0.8
- Vegetarianism	-0.03	-0.04	(-0.12, 0.04)	-0.93	0.4
- Vitamin use	0.14	0.11	(0.06, 0.16)	4.2	<0.001
Pregnancy related					
- Parity	-0.008	-0.006	(-0.05, 0.04)	-0.23	0.8
Haemodilutional					
- Haematocrit (%)	0.12	1.66	(0.76, 2.56)	3.59	<0.001

* Denotes variable transformed using natural logs (allowing β coefficients to be interpreted in terms of percentage change)

#### Folate associations

In a multiple regression analysis examining the associations between folate and obesity-related factors, the pattern was less clear than seen with B12. BMI was no longer an independent predictor of low folate. The obesity-associated factor explaining most of the variation in folate was AST, with triglycerides and HDL also showing weak independent associations (Table 7)).

**Table 7:** Multiple regression analysis to demonstrate the relationships between BMI, BMI associated factors, confounding variables and haemodilution on serum vitamin folate.

Factors associated with serum folate*	Partial R	Beta	95% CI	t	p
BMI (kg/m^2^)*	-0.06	-0.26	(-0.54, 0.02)	-1.80	0.07
BMI associated factors					
- HOMAR	-0.05	-0.07	(-0.17, 0.03)	-1.45	0.2
- Triglycerides (mmol/L)	0.07	0.12	(0.003, 0.239	2.01	0.05
- HDL cholesterol	0.07	0.09	(0.009, 0.17)	2.17	0.03
- AST (mmol/L)*	0.16	0.36	(0.21, 0.51)	4.7	<0.001
- FPG	0.006	0.01	(-0.10, 0.20)	0.18	0.9
Social/demographic					
- Age	0.13	0.02	(0.008, 0.02)	3.82	<0.001
- Townsend score	-0.02	-0.003	(-0.02, 0.008)	-0.54	0.6
- Smoking	-0.08	-0.13	(-0.25, -0.02)	-2.26	0.02
- Vegetarianism	0.18	0.36	(0.23, 0.49)	5.45	<0.001
- Vitamin use	0.41	0.55	(0.47, 0.63)	13.30	<0.001
Pregnancy related					
- Parity	0.09	0.11	(0.031, 0.18)	2.77	0.006
Haemodilutional					
- Haematocrit (%)	0.04	0.94	(-0.49, 2.38)	1.29	0.2

* Denotes variable transformed using natural logs (allowing β coefficients to be interpreted in terms of percentage change)

### Maternal BMI and interaction of folate and B12

**Fig 1:**
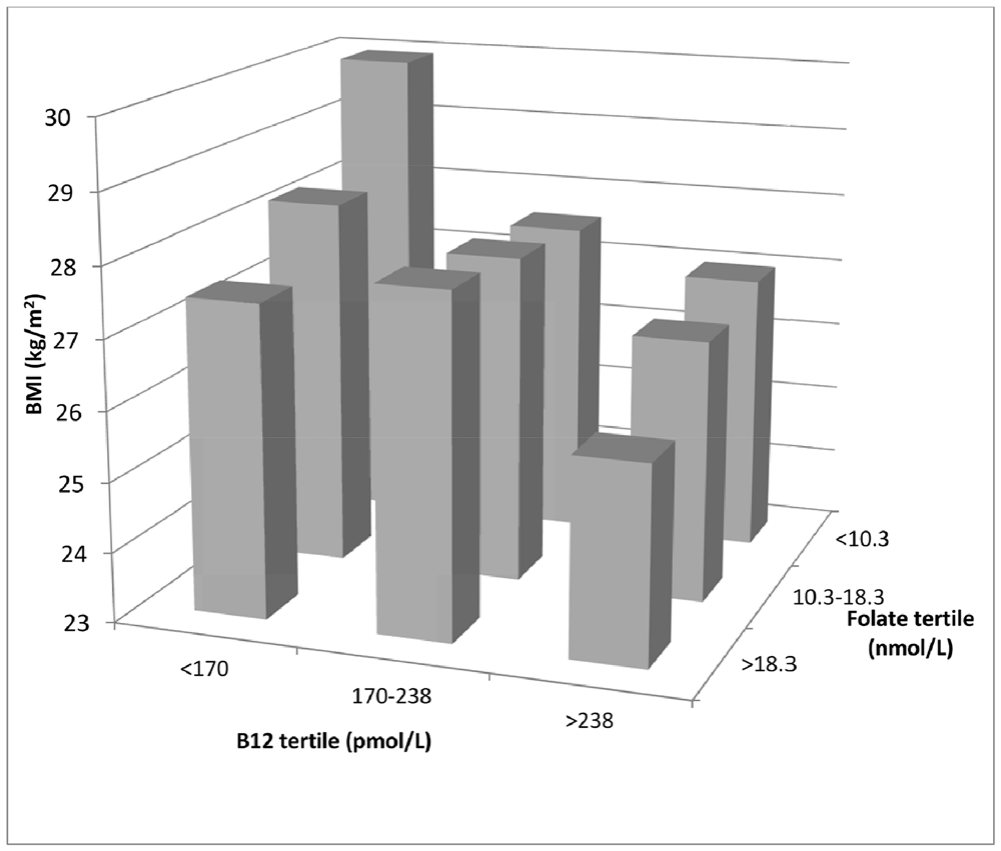
Association of maternal BMI with vitamin B12 and folate concentrations (tertiles). Bars represent geometric mean BMI for each group.

We also investigated the association of maternal BMI with vitamin B12 and folate concentrations simultaneously. Women with the lowest B12 and folate concentrations had the highest BMI and those with highest B12 and folate concentrations had the lowest BMI (29.9 vs 25.8 kg/m^2^, p<0.001) (Fig 1).

## Discussion

In this first cross-sectional study in the UK we identified that higher BMI is associated with lower circulating B12 and folate during pregnancy in a non-diabetic Caucasian British population at 28 weeks gestation.

The inverse association of vitamin B12 in pregnancy with obesity and insulin resistance is in keeping with the previous study from India[4]. The British women in our cohort had higher serum B12 levels and lower folate concentrations at 28 weeks gestation (201 vs 162 pmol/l and 14 vs 35nmol/l respectively) compared to Indian mothers when measured in the identical assay. This resulted in fewer with low B12 (<150 pmol/L) (20% vs 43%) but more with low folate (<7nmol/L) (15% vs 4%). The British mothers were non-diabetic, heavier, older and predominantly non-vegetarian. Despite these differences, we observed that lower circulating B12 was associated with higher BMI and insulin resistance, suggesting that these associations are more general and not only restricted to the Indian population with its vegetarian dietary habits.

We have shown that the association of higher BMI with lower B12 is unlikely to be the result of the potential confounding effects of demographic, lifestyle, pregnancy, or haemodilutional factors, suggesting it could be the result of obesity in pregnancy itself. We explored the associations of BMI related biochemical indices with vitamin B12 and folate. BMI remains an independent predictor of circulating B12, as do HOMA-R, triglycerides and AST. These indices are markers of adiposity/body fat metabolism and support the hypothesis that altered maternal body fat distribution/metabolism during pregnancy may be implicated in determining circulating concentrations of micronutrients in pregnancy.

Vitamin B12 and folate are essential for cell growth and maintenance, and play crucial roles during fetal development. Our findings suggest that increasing maternal obesity in mothers may result in a reduction in circulating concentrations of essential micronutrients needed by both mother and her fetus. The increasing evidence of the role of one-carbon metabolism (important for the synthesis of nucleic acids and DNA methylation) in fetal growth and programming of non-communicable diseases has been summarised recently[10]. The fetuses of obese mothers are known to be at increased risk of a range of malformations, including neural tube defects and cardiovascular abnormalities. Our findings raise the possibility that these could be mediated by low levels of vitamin B12 and folate which are known to be involved in these processes[11], and if confirmed would strengthen the evidence in favour of special needs of vitamin supplementation for obese women planning to become pregnant. To confirm this would require larger studies, including interventional studies, measuring B12 and folate in the first trimester.

Our study has several limitations: it is a cross sectional study with measures of B12 and folate at one time point only, therefore our findings may not be representative of the crucial early stages of pregnancy or late trimester. We suggest future studies should include longitudinal sequential sampling from early gestation. Residual confounding by socio-economic status and dietary factors is possible because women with lower B12 and folate concentrations were younger, more obese and more socially deprived. A recent review suggested that the micronutrient deficiencies associated with overweight and obesity may result from increased intake of relatively cheap, energy dense but nutrient poor foods[12].

We have identified associations but these do not define a causal relationship or its direction. These issues could be investigated further by the use of animal models, by future RCT studies on the effects of micronutrition on pregnancy and gestational development, or by using the technique of Mendelian randomisation employing genetic variants associated with vitamin B12 and folate.

In conclusion, our study has replicated the previous Indian findings of associations between lower serum B12 and higher obesity and insulin resistance during pregnancy in a non-diabetic White British population. These findings may have important implications for fetal and maternal health in obese pregnancies.
